# Long-term mortality in HIV patients virally suppressed for more than three years with incomplete CD4 recovery: A cohort study

**DOI:** 10.1186/1471-2334-10-318

**Published:** 2010-11-02

**Authors:** Frederik N Engsig, Jan Gerstoft, Gitte Kronborg, Carsten S Larsen, Gitte Pedersen, Birgit Røge, Janne Jensen, Lars N Nielsen, Niels Obel

**Affiliations:** 1 Department of Infectious Diseases, Copenhagen University Hospital, Rigshospitalet, Blegdamsvej 9, 2100 Copenhagen Ø, Denmark; 2 Department of Infectious Diseases, Copenhagen University Hospital, Hvidovre Hospital, Kettegårds Allé 30, 2650 Hvidovre, Denmark; 3 Department of Infectious Diseases, Aarhus University Hospital, Skejby Sygehus, Brendstrupgårdsvej, DK8200 Aarhus N, Denmark; 4 Department of Infectious Diseases, Aalborg University Hospital, 9000 Aalborg, Denmark; 5 Department of Infectious Diseases, Odense University Hospital, Sønderbouldevard 65, 5000 Odense C, Denmark; 6 Department of Infectious Diseases, Fredericia and Kolding Sygehus, Skovvangen 2-8, 6000 Kolding, Denmark; 7 Department of Infectious Diseases - Hillerød, Hillerød Sygehus, Dyrehavevej 29, Hillerød 3400, Denmark

## Abstract

**Background:**

The mortality in patients with persistent low CD4 count despite several years of HAART with sustained viral suppression is poorly documented. We aimed to identify predictors for inadequate CD4 cell recovery and estimate mortality in patients with low CD4 count but otherwise successful HAART.

**Method:**

In a nationwide cohort of HIV patients we identified all individuals who started HAART before 1 January 2005 with CD4 cell count ≤ 200 cells/μL and experienced three years with sustained viral suppression. Patients were categorized according to CD4 cell count after the three years suppressed period (≤ 200 cells/μL; immunological non-responders (INRs), >200 cells/μL; immunological responders (IRs)). We used logistic regression and Kaplan-Meier analysis to estimated risk factors and mortality for INRs compared to IRs.

**Results:**

We identified 55 INRs and 236 IRs. In adjusted analysis age > 40 years and > one year from first CD4 cell count ≤ 200 cells/μL to start of the virologically suppressed period were associated with increased risk of INR. INRs had substantially higher mortality compared to IRs. The excess mortality was mainly seen in the INR group with > one year of immunological suppression prior to viral suppression and injection drug users (IDUs).

**Conclusion:**

Age and prolonged periods of immune deficiency prior to successful HAART are risk factors for incomplete CD4 cell recovery. INRs have substantially increased long-term mortality mainly associated with prolonged immunological suppression prior to viral suppression and IDU.

## Background

The introduction of HAART has decreased morbidity and mortality in HIV patients due to viral load (VL) suppression and immunological recovery [1]. Still, the immunological reconstitution after HAART initiation varies depending on the pre-HAART level of immunodeficiency [2-7]. Several studies have shown that patients with successful virological response to HAART and incomplete initial CD4 recovery have increased mortality [5,8,9]. However, the outcome is poorly documented for patients with pesistent low CD4 count despite several years of HAART with sustained VL suppression.

Within a nationwide cohort of HIV infected patients we identified all individuals who were on stable HAART, had been virally suppressed for more than three years and had a CD4 cell count ≤ 200 cells/μL prior to the virally suppressed period. In this population we identified immunologic non-responders who did not rise to a CD4 cell count > 200 cells/μL after the three years of sustained VL suppression. We aimed to identify predictors for inadequate CD4 cell recovery and to determine the mortality in the immunological non-responders compared to those with an adequate CD4 cell response.

## Methods

The study was performed as a nationwide cohort study. In the first part of the study we estimated risk factors for immunological non-response (CD4 cell count ≤ 200 cells/μL after three years of VL suppression). In the second part of the study we estimated mortality in immunological non-responders (INRs) versus immunological responders (IRs).

### Setting

Denmark had a population of 5.5 million as of 31 December 2008, with an estimated HIV prevalence of approximately 0.07% in the adult population [10,11]. Patients with HIV infection are treated in one of the country's eight specialized medical centres, where they are seen on an outpatient basis at intended intervals of 12 weeks. Antiretroviral treatment is provided free of charge to all HIV-infected residents of Denmark. The national criteria for initiating HAART have been described previously [12].

### Data sources

The Danish HIV Cohort study (DHCS), described in details elsewhere, is a population-based prospective nationwide cohort study of all HIV-infected individuals 16 years or older at diagnosis and who are treated at Danish HIV centres after 1 January 1995 [13]. December 31, 2008 the cohort included 5206 Danish residents. Patients are consecutively enrolled, and multiple registrations are avoided through the use of a unique 10-digit civil registration number assigned to all individuals in Denmark at birth or upon immigration. Data are updated yearly and includes demographics, date of HIV infection, AIDS defining events, date and cause of death and antiretroviral treatment. CD4 cell counts and HIV-RNA measurements are extracted electronically from laboratory data files. After 1 January 2000 all viral load (VL) analyses in Denmark were designed to measure VL below 50 copies/ml. Before that period lower limit of detection were in some centres 199 copies/ml and 399 copies/ml and these values are in the present study considered being below 50 copies/ml. Patients are intended tested yearly for hepatitis C antibodies, and if positive further tested for hepatitis C RNA.

Primary causes of death were obtained from The Danish Civil Registration System and The Danish Register of Causes of Death [14]. Data on date of cancer diagnosis were obtained from The Danish Cancer Registry and all patients diagnosed with cancer in the suppressed period or 10 years previous to this were identified [15].

### Study population

From DHCS we identified all HIV-1 positive patients, who 1) started HAART before 1 January 2005, 2) had a CD4 cell count ≤ 200 cells/μl at start of HAART, 3) had a VL < 50 copies/ml for more than three consecutive years before 1 January 2008, 4) had no intervals of more than seven months between VL tests in the suppressed period, and 5) had a CD4 cell count ≤ 200 cells/μl at start of the virally suppressed period.

Patients were defined as IRs in case the first CD4 cell count measured after 3 years of viral suppression was > 200 cells/μL and INRs in case the CD4 count was ≤ 200 cells/μL. Mortality was also stratified for more differentiated CD4 cell strata after 3 years of viral suppression (>200 cells/μL and ≤ 350 cells/μL, > 350 cells/μL and ≤ 500 cells/μL and >500 cells/μL) but the prognosis differed little for all strata above 200 cells/μL why we chose to pool all CD4 responses above 200 cells/μL in one group (data not shown).

### Statistics

Differences in characteristics between groups were evaluated by the χ^2 ^test, Fisher's exact test and Kruskal-Wallis Test when appropriate.

We used binary logistic regression in order to identify predictors for immunological non-response. The following covariates were included in the model: Gender, age at start of the suppressed period (≤ 40 years vs. > 40 years) (The cut point was based on the median age; IRs; 37.6 years (IQR; 32.1 - 45.3) and INRs; 42.6 years (IQR; 36.1 - 51.3)), race (Caucasian vs. non-Caucasian), route of HIV infection (men who have sex with men (MSM) vs. heterosexually vs. injection drug user (IDU) vs. other), chronic HCV infection (positive vs. negative PCR for HCV RNA), cancer prior to start of the suppressed period, one or more AIDS defining event before start of suppressed period, HIV diagnose before 1 January 1995, nadir CD4 cell count < 50 cells/μL and > one year from first CD4 ≤ 200 cells/μL to start of the virologically suppressed period (The cut point of one year was based on the median time from first CD4 cell count ≤ 200 to start of the suppressed period; IRs; 0.7 year (IQR; 0.3 - 2.2) and INRs; 1.5 years (IQR; 0.4 - 3.2)). Selection of potential confounders was performed using the "change in estimate" method with age and gender forced into the model [16].

Index date was defined as date of first CD4 cell count measurement after three years of sustained viral suppression. We calculated person-years at risk from index date until death, 1 January 2008, emigration or lost to follow-up, whichever came first. We used Kaplan Meier analysis to construct survival curves for INRs vs. IRs and further stratified these by time from first CD4 measurement ≤ 200 cells/μL to start of the virologically suppressed period (≤ one year vs. > one year). Cox regression analyses were used to estimate mortality rate ratios (MRR). We further calculated MRRs stratified by cancer (no cancer diagnosed prior to index date vs. cancer diagnosed prior to index date), previous AIDS defining event (none vs. > one or more AIDS defining events prior to index date) and time from first CD4 measurement ≤ 200 cells/μL to start of the virologically suppressed period (≤ one year vs. > one year). The estimates were adjusted for gender and age.

In a robustness analysis we used a cut-off value of 500 copies/ml in the virologically suppressed period to test the impact of VL cut-off on our estimates.

### VL and CD4 cell response in the observation period

Using a previously described method [13] we grouped all CD4 cell measurements and VL tests in the observation period in 12-weeks intervals and calculated the proportion of CD4 measurements ≤ 200 cells/μL and VL tests < 50 copies/ml for both IRs and INRs up to six years after index date.

The study was approved by the Danish Data Protection Agency (Denmark has no Institutional review boards). Data in the database is not publicly available. SPSS statistical software, Version 15.0 (Norusis; SPSS Inc., Chicago, Illinois, USA) and R software, version 2.8.1, was used for data analysis.

## Results

**Figure 1:**
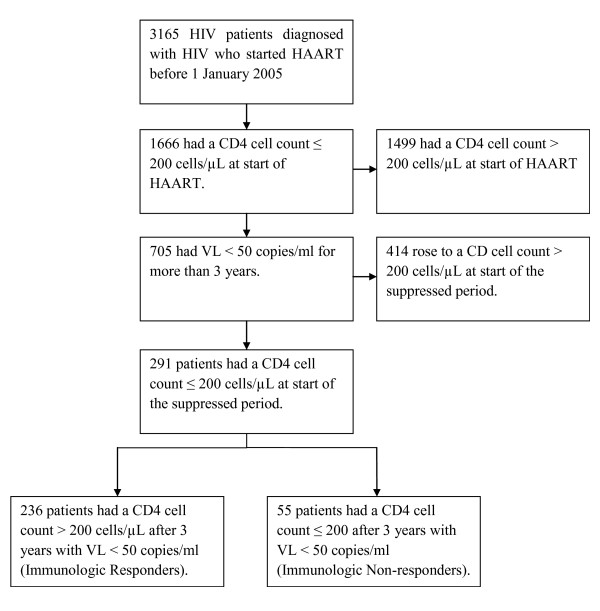
Study flow chart.

In The Danish HIV Cohort Study 3165 patients started HAART before 1 January 2005 (figure 1).291 study subjects fulfilled the inclusion criteria and were followed for 1373 person-years during which 7 patients (2.4%) emigrated. 227 (78.0%) of the patients were males and 218 (74.9%) were Caucasians. Median time between VL tests in the suppressed period was 88 days (IQR; 59 - 98).

After three years of sustained VL 236 (81.1%) patients reached a CD4 cell count above 200 cells/μl (IRs) and 55 (18.9%) of the HIV infected patients did not (INRs). Characteristics of IRs and INRs are shown in table 1.

**Table 1:** Characteristics and demographics of immunological responders and non-responders.

Characteristics	Immunologic responders	Immunologic non-responders	P
	N = 236	N = 55	
Male gender	179 (75.8)	48 (87.3)	0.065
Age at time of index date, median (IQR), years	37.6 (32.1 - 45.3)	42.6 (36.1 - 51.3)	<0.001
Caucasians	171 (72.5)	47 (85.5)	0.045
Route of HIV infection			0.059
Men who have sex with men	97 (41.1)	29 (52.7)	
Heterosexually infected	98 (41.5)	14 (25.5)	
Injection drug user	14 (5.9)	7 (12.7)	
Other	26 (11.8)	3 (6.1)	
Chronic HCV infection	20 (8.5)	8 (14.5)	0.169
Cancer within 13 years prior to index date	27 (11.4)	10 (18.2)	0.177
One or more AIDS defining events prior to index date	114 (48.3)	24 (43.6)	0.532
One or more AIDS defining events after index date	3 (1.3)	2 (3.6)	0.240
Diagnosed with HIV before 1 January 1995	94 (49.8)	27 (49.1)	0.210
Treated with zidofovir prior to index date	219 (92.8)	54 (98.2)	0.465
Nadir CD4 cell count, median (IQR), cells/μL	40 (10 - 82)	40 (15 - 57)	0.289
More than one year from first CD4 cell count ≤ 200 cells/μL to start of the virologically suppressed period*	96 (40.7)	32 (58.2)	0.019
Time from start of HAART to start of the virologically suppressed period, median (IQR), years	0.4 (0.2 - 1.1)	0.4 (0.3 - 1.3)	0.518

* Median time from first CD4 cell count ≤ 200 cells/μL to start of the virologically suppressed period was 0.7 year (IQR; 0.3 - 2.2) for IRs and 1.5 years (IQR; 0.4 - 3.2) for INRs.

In un-adjusted analysis age, Caucasian race and time from first CD4 cell count ≤ 200 cells/μL to start of the virologically suppressed period were associated with immunological non-response (table 2). However, after having adjusted for potential confounders only age and time from first CD4 cell count ≤ 200 cells/μL to start of the virologically suppressed period remained associated with increased risk of being INR (table 2).

**Table 2:** Odds ratios (OR) for immunological non-response in patients on successful HAART for more than three years.

Characteristics	OR Un-adjusted (95% CI*)	OR, Adjusted ** (95% CI)
Male gender	2.18 (0.94 - 5.09)	1.73 (0.73 - 4.14)
More than 40 years old at index date	2.53 (1.390 - 4.63)	2.32 (1.25 - 4.29)
Caucasians	2.23 (1.01 - 4.98)	1.09 (0.36 - 3.36)
Route of HIV infection		
Men who have sex with men	1.0	
Heterosexually infected	0.48 (0.24 - 0.96)	0.73 (0.30 - 1.78)
Injection drug user	1.67 (0.63 - 4.54)	3.06 (0.98 - 9.52)
Other	0.62 (0.22 - 1.75)	0.63 (0.20 - 1.96)
Chronic HCV infection	1.84 (0.76 - 4.43)	2.11 (0.79 - 5.61)
Cancer within 13 years prior to index date	1.72 (0.78 - 3.81)	1.47 (0.63 - 3.44)
One or more AIDS defining events prior to index date	0.83 (0.46 - 1.50)	0.84 (0.46 - 1.53)
Diagnosed with HIV before 1 January 1995	1.46 (0.81 - 2.63)	1.43 (0.72 - 2.86)
Nadir CD4 cell count less than 50 cells/μL	1.59 (0.86 - 2.96)	1.06 (0.43 - 1.75)
More than one year from first CD4 ≤ 200 cells/μL to start of the virologically suppressed period	2.03 (1.12 - 3.68)	2.28 (1.18 - 4.43)

*Confidence interval (95%CI).

**All estimates adjusted for age and gender.

The distribution of drug classes included in the last HAART regimen prior to index date did not differ between IRs and INRs (Additional file 1).

**Figure 2:**
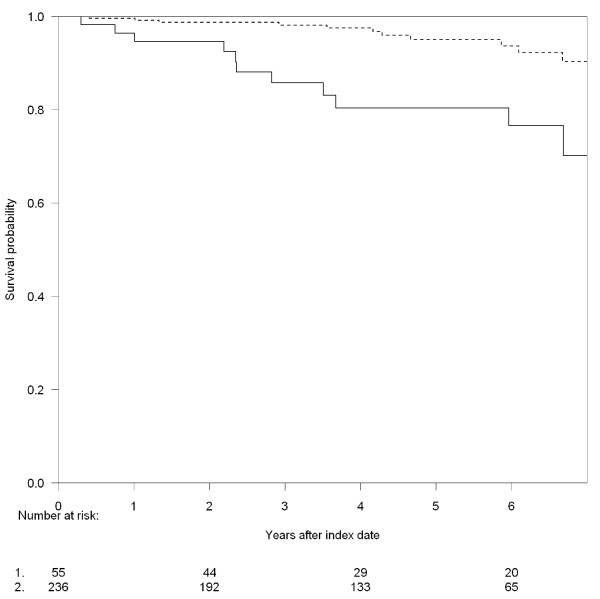
**Kaplan-Meier curves for overall survival according to immunologic response at index date: 1. **CD4 cell count ≥ 200 cells/μL (broken line), 2. CD4 cell count < 200 cells/μL (full line). Index date was defined at the date of first CD4 measurement after three years of viral suppression (VL < 50 HIV-RNA copies/ml).

**Figure 3:**
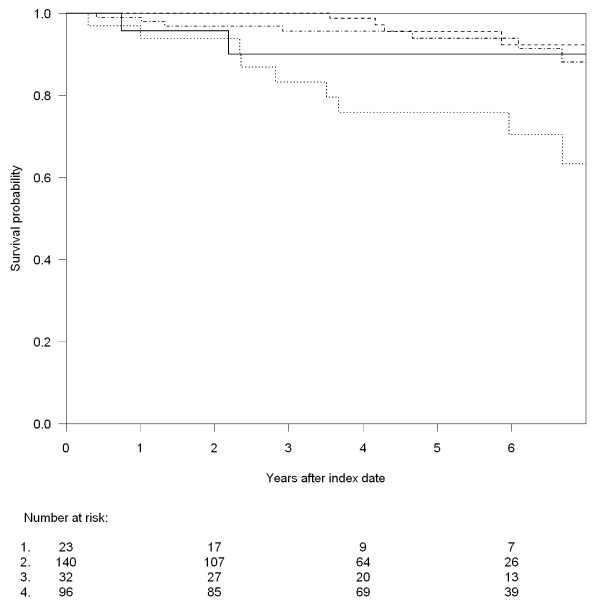
**Kaplan-Meier curves for overall survival according to CD4 count at index date and stratified by time from first CD4 cell count < 200 cells/μL to start of the virally suppressed period: **1) CD4 ≥ 200 cells/μL and ≤ one year (full line), 2) CD4 ≥ 200 cells/μL and > one year (bar line). 3) CD4 < 200 cells/μL and ≤ one year (dot and bar line). 4) CD4 < 200 cells/μL and > one year (dot line).

A total of 22 (7.6%) patients died in the observation period, 11 (20.0%) in the INR group and 11 (4.7%) in the IR group. As shown in figure 2, INRs had substantially higher mortality compared to IRs. Unadjusted MRR for INRs compared to IRs was 4.2 (95%CI; 1.8 - 9.6) and 3.4 (95%CI; 1.4 - 8.0) after adjustment for age and gender. Figure 3 shows the mortality for IRs vs. INRs stratified by time from first CD4 measurement ≤ 200 cells/μL to start of of the virologically suppressed period and from this analysis it is seen, that the main excess mortality was observed in the INRs with more than one year of immunological suppression prior to the virologically suppressed period. Of importance, the excess mortality was observed up to 6.5 years after initiation of virologically successful HAART. The INRs did not experience substantially more AIDS defining events in the observation period (3.6% vs. 1.3%).

128 patients (44.0%) had more than one year from first CD4 measurement ≤ 200 cells/μL to start of the virologically suppressed period. The un-adjusted MRR among these 128 patients was 3.9 (95%CI; 1.5 - 10.6) and after adjustment for age and gender it was 3.6 (95%CI; 1.3 - 9.7). When excluding these patients from the analysis the unadjusted MRR was reduced to 3.1 (95%CI; 0.6 - 17.7) and after adjustment for age and gender it was 1.8 (95%CI; 0.3 - 10.2). 90 (70.3%) of the 128 patients who had more than one year of immunological suppression prior to the virologically suppressed period were diagnosed before 1995. When excluding IDUs from our analysis the unadjusted MRR was 2.5 (95%CI; 0.9 - 7.8) and the adjusted MRR was reduced to 1.8 (95%CI; 0.6 - 5.1). The un-adjusted MRR calculated exclusively for IDUs was 12.7 (95%CI; 1.5 - 109.9) and 9.8 ((95%CI; 1.1 - 86.1) after adjustment for age and gender. Exclusion of patients with previous cancer or AIDS defining event, respectively, did not change the estimates substantially. Only one death in each group (one PML and one cryptococcal meningitis) appeared to be a classical HIV related cause of death. Other causes of death were: cancer (INR: 3, IR: 2), sudden death (INR: 3, IR 3), liver related (INR:2, IR: 1), suicide (INR: 1, IR: 1), opiate overdose (INR: 1, IR: 0), pneumonia (INR:2, IR:0) and cachexia (not HIV related) (INR: 0, IR: 1).

80% of the INRs achieved a CD4 cell count > 200 cells/μL after six years of observation (i.e. more than nine years after starting HAART). However, only 20 (35.1%) of the INRs were still under observation six years after study inclusion. After six years of observation, 95.5% of the whole study population who were still alive had fully suppressed VL.

When using a cut-off value of 500 copies/ml for viral suppression, 445 study subjects (372 IRs and 73 INRs) were identified, of whom 18 (4.5%) patients in the IR group and 15 in the INR group (20.5%) died during follow-up. In the un-adjusted analysis age, Caucasian race, IDU, time from first CD4 cell count ≤ 200 cells/μL to start of the virologically suppressed period and CD4 cell count at start of the suppressed period were associated with immunological non-response. However, after having adjusted for potential confounders only age, IDU and CD4 cell count at start of the suppressed period remained associated with increased risk of being INR. MRRs were 4.5 (95%CI; 2.3 - 9.0) and 3.69 (95%CI; 1.8 - 7.4) when adjusted for gender and age.

## Discussion and Conclusions

In a population of HIV patients with initial low CD4 count we found that almost one out of five was INR after three years of successful HAART. Older age and more than one year of severe immune deficiency prior to start of sustained VL were associated with INR. The patients with insufficient immunological response had substantially increased long term mortality. The increased mortality was mainly seen in patients with more than one year of immunological deficiency prior to the period of sustained VL suppression and IDUs.

The major strength of this study is the identification of a homogeneous group with stringent viral suppression, essentially eliminating the possibility that incomplete VL suppression explains our findings. The study had a nationwide design with long and almost complete follow-up including data on cancer as well as HCV status.

A limitation of the study is the small study population (in part caused by the stringent definitions of INRs and IRs) and the small number of outcomes, which did not allow us to extend the logistic regressions analysis or to calculate cause specific rate ratios of death. The study population encompasses a highly selected group of patients surviving more than three years in spite of low initial CD4 cell count, and a healthy survivor effect may lead to underestimation of the excess mortality in the INRs. Still, by using an extended virally suppressed period we allowed the patients to reach a steadier CD4 plateau without the interference of CD4 cell redistribution as seen after HAART start. HCV testing in the cohort is "intended" and may only be performed yearly on known high risk groups in the cohort e.g IDU why chronic HCV may be underestimated in the cohort.

19% of the study patients did not have an adequate immunological response to HAART. Our results corresponds to the findings of other studies who found a negative impact of low initial CD4 cell count on long-term CD4 recovery [2,3,6,17-19].

INRs were significantly older than the IRs and age predicted inadequate CD4 cell recovery which corresponds to the findings of several other studies [3,5,9,19,20]. In contrast to other studies, we did not find a statistically significant association between IDU or chronic HCV infection and INR [3,21]. However, when using a cut-off value of 500 copies/ml, IDU was associated with INR. This is probably due to the inclusion of a larger number of study patients with these characteristics when allowing for viral blips. Nadir CD4 cell count did not differ between the two groups, presumably reflecting that immunological responders without a low nadir were able to escape above the 200 cells/μL threshold before full suppression was observed. More INRs had more than one year from first CD4 cell count ≤ 200 cells/μL to start of the virologically suppressed period, indicating that they had a longer period of immunological suppression than the IRs and this factor was also associated with incomplete CD4 cell recovery. Defect bone marrow function and decreased thymus activity are major suspects in delayed CD4 cell recovery in HIV patients with severe immunodeficiency [4,22-25]. To our knowledge no studies have been made on predictors for bone marrow function in HIV infected patients, but our results are in accordance with the findings of Dion et al, who found that both older age and prolonged immunological suppression were associated with decreased thymic activity in virally suppressed HIV patients resulting in slower CD4 recovery [26].

The excess mortality seen among the INRs was mainly related to prolonged immunological suppression prior to successful HAART and IDU. The median time from start of HAART to start of the virologically suppressed period was less than half a year. A longer period of immunological suppression prior to the three years of sustained VL suppression is therefore explained by delayed initiation of HAART and not poor compliance. This is supported by the fact that most of the patients with more than one year of immunological suppression before start of the virologically suppressed period were diagnosed with HIV before 1995 when treatment options were scarce. As many of the INRs who died were patients who initiated HAART in the mid-nineties it could be hypothesized that resistance played a role, but the stringent design with full VL suppression eliminates that possibility. We find it unlikely that the late death of these patients were directly related to the toxicity of the antiretroviral agents, but cannot rule out that toxicity should interact with the immune reconstitution. The increased mortality in patients with delayed initiation of HAART is well documented [27] and the contribution of persistent immunodeficiency to the development of e.g. cancer has also been observed by others [28]. It may be speculated that intermittent viraemias in this setting would increase mortality but the estimate remained unchanged in our sensitivity analysis using a cut-off value of 500 copies/ml for viral suppression, suggesting that either the impact of low-level replication on death and immune reconstitution is limited or that the majority of bleeps below 500 remains laboratory artefacts.

We only encountered two AIDS related deaths suggesting that these are rare after prolonged HAART even without adequate CD4 recovery. Thus, it seems that the INRs are not immune-suppressed in the classical sense where a low CD4 count leads to AIDS defining diseases and eventually death. Still, we cannot rule out that there is a biological cause of the increased mortality in immunological non-responders related to immunodeficiency and that our study simply do not have power enough to demonstrate an association.

The risk factors for incomplete CD4 cell recovery and increased mortality points toward earlier diagnosis of HIV as the main prophylactic measure. Also, the increased mortality in these patients calls for increased concern in terms of treatment and screening for co-morbidity.

We conclude that age and prolonged immunological suppression are risk factors for incomplete CD4 cell recovery in patients with otherwise successful HAART. Patients with insufficient immunological response have substantially increased long-term mortality compared to IRs, but the increased mortality is mainly associated with prolonged immunological suppression prior to VL suppression and IDU. We therefore presume that in the modern HAART era where patients are started early on HAART the prevalence of insufficient CD4 recovery will decrease substantially.

## Competing interests

Potential competing of interest: N Obel has received research funding from Roche, Bristol-Myers Squibb, Merck Sharp & Dohme, GlaxoSmithKline, Abbott, Boehringer Ingelheim, Janssen-Cilag and Swedish Orphan. F Engsig has received research funding from Merck Sharp & Dohme. J Gerstoft has received research funding from Abbott, Roche, Bristol-Myers Squibb, Merck Sharp & Dohme, Pharmasia, GlaxoSmithKline, Swedish Orphan and Boehringer Ingelheim.

## Financial support

The study was financed by The Research Foundation at Copenhagen University Hospital, Rigshospitalet and Faculty of Health Science, Copenhagen University. The funders had no role in the study design; in the collection, management, analysis, and interpretation of data; in the preparation, review, or approval of the manuscript, or in the decision to submit the article for publication. The researchers are independent from the funders.

## Authors' contributions

The authors contributions are the following: FNE (MD) contributed with study design, data collection, data analysis, interpretation of findings and writing of the manuscript. JG (MD, Professor, DrMedSc) contributed with study design, data collection, interpretation of findings and critical edit of the manuscript. GK (MD, associate professor, DrMedSc), CSL (MD, DrMedSc), GP (MD), BR (MD, PhD), JJ (MD) and LNN (MD)contributed with data collection, study design, interpretation of findings and critical edit of the manuscript. NO (MD, DrMedSc) contributed with data collection, study design, critical review of data analyses, interpretation of findings and critical edit of the manuscript. All authors read and approved the final manuscript.

## Pre-publication history

The pre-publication history for this paper can be accessed here:

http://www.biomedcentral.com/1471-2334/10/318/prepub

## Supplementary Material

Additional file 1 **Table S1**. Drugs included in the last HAART regimen prior to index date. Click here for file
