# Supplementary material for: Long-term mortality in HIV patients virally suppressed for more than three years with incomplete CD4 recovery: A cohort study
Source: BMC Infect Dis. 2010 Nov 2;10:318. doi: 10.1186/1471-2334-10-318 (PMC2988053; doi:10.1186/1471-2334-10-318)
Supplement: Additional file 1 — Table S1. Drugs included in the last HAART regimen prior to index date. [file 1471-2334-10-318-S1.DOC]

| **Drugs included in the last HAART regimen prior to index date** | **IR**  **N (%)** | **INR**  **N (%)** |
| --- | --- | --- |
| NRTI | 234 (99.2) | 55 (100%) |
| 1 NRTI | 8 (3.4) | 3 (5.5) |
| 2 NRTI | 215 (91.1) | 49 (89.1) |
| 3 NRTI | 11 (4.7) | 3 (5.5) |
| NNRTI | 99 (41.9) | 21 (38.2) |
| PI | 158 (66.9) | 38 (69.1) |
| Boosted PI | 60 (38.0) | 11 (28.9) |
| Non-boost PI | 98 (62.0) | 27 (71.1) |
| Maraviroc, etravirine, darunavir or T20 | - | - |

**Additional table 1.**
